# Multiparametric MRI with MR elastography findings in patients with sinusoidal obstruction syndrome after oxaliplatin-based chemotherapy

**DOI:** 10.1186/s13244-022-01281-w

**Published:** 2022-09-05

**Authors:** Ahmet Poker, Musturay Karcaaltıncaba, Mustafa N. Ozmen, Ali D. Karaosmanoğlu, Ahmet G. Erdemir, Osman Ocal, Deniz Akata, Ilkay S. Idilman

**Affiliations:** Department of Radiology, Hacettepe University School of Medicine, Ankara, 06100 Turkey

**Keywords:** Sinusoidal obstruction syndrome, MR elastography, MRI mapping

## Abstract

**Objective:**

To evaluate the magnetic resonance elastography (MRE)-derived liver stiffness measurement (LSM), T1 and T2 relaxation times, and hepatobiliary phase images in patients, who developed sinusoidal obstruction syndrome (SOS) after oxaliplatin-based chemotherapy.

**Methods:**

Thirty-four patients (M/F:22/12) who underwent liver MRI-MRE and received oxaliplatin for colorectal, gastric, and pancreas cancer were included in the study. SOS was diagnosed by Gd-EOB-DTPA-enhanced MRI in 18 patients. MRE-LSM and T1–T2 maps were evaluated. Patients with SOS were grouped according to the amount of reticular hypointensity on the hepatobiliary phase images.

**Results:**

The mean MRE-LSM in the patients with SOS was 3.14 ± 0.45 kPa, and the control group was 2.6 ± 0.5 kPa (*p* = 0.01). The mean-corrected T1 (cT1) relaxation time was 1181 ± 151 ms in the SOS group and 1032 ± 129 ms in the control group (*p* = 0.005). The mean T2 relaxation time was 50.29 ± 3.6 ms in the SOS group and 44 ± 3.9 ms in the control group (*p* = 0.01). Parenchymal stiffness values were 2.8 ± 0.22 kPa, 3 ± 0.33 kPa, and 3.65 ± 0.28 kPa in patients with mild, moderate, and advanced SOS findings, respectively (*p* = 0.002). Although cT1 and T2 relaxation times increased with increasing SOS severity, no statistical significance was found.

**Conclusions:**

We observed increased MRE-LSM in patients with SOS after chemotherapy compared to control group. T1 and T2 relaxation times were also useful in diagnosing SOS but were found inadequate in determining SOS severity. MRE is effective in diagnosing SOS and determining SOS severity in patients who cannot receive contrast agents, and it may be useful in the follow-up evaluation of these patients.

## Key points

- Sinusoidal obstruction syndrome (SOS) has been frequently associated with oxaliplatin-based chemotherapy.
- Increased MRE-LSM and cT1–T2 relaxation times in patients with SOS after oxaliplatin-based chemotherapy were observed.
- A good correlation with MRE-LSM and SOS severity according to the extent of reticular hypointensity was observed.

## Introduction

Hepatic sinusoidal obstruction syndrome (SOS) is an obliterative venulitis of the small hepatic veins with a high mortality risk in its severe forms [1]. SOS, also known as veno-occlusive disease, can develop in many conditions, such as after hematopoietic stem cell transplantation, after the use of oxaliplatin-containing adjuvant or neoadjuvant chemotherapy, and in patients using herbal medicines containing pyrrolizidine alkaloids [1, 2]. Oxaliplatin-containing chemotherapy protocols are used to treat metastatic colorectal carcinoma, advanced gastric carcinoma, and adenocarcinoma of the pancreas. SOS leads to impaired liver function, resulting in increased morbidity and mortality after liver resection [3]. Moreover, advanced stage SOS has been found to cause inadequate effect of chemotherapeutic agents on the tumor in colorectal cancer patients [4]. Weight gain, painful hepatomegaly, and jaundice represent the classic triad of SOS in a patient with stem cell transplantation and has a history of using a plant containing pyrrolizidine alkaloids [5]. However, subtle or no clinical findings may occur in some patients with oxaliplatin-induced SOS, which causes difficulties in the diagnosis of SOS in such patients [6]. Reticular hypointensity observed on hepatobiliary phase images of gadoxetate/gadoxetic acid (Gd-EOB-DTPA)-enhanced MRI examination in patients treated with oxaliplatin is a specific finding for the diagnosis of SOS [6, 7]. However, it can be challenging to use contrast agent in patients with kidney dysfunction.

In clinical practice, noninvasive imaging tools such as proton density fat fraction (PDFF), T2*, T1–T2 mapping, and MR elastography are frequently used multiparametric MRI methods. It provides quantitative characterization of tissue composition, inflammation, fibrosis, fat, and iron deposits in the liver [8, 9]. Magnetic resonance elastography (MRE) is a phase-contrast MRI technique that characterizes the elastic properties of tissues by analyzing the mechanical wave propagation in the tissue [10]. It has been shown to be an accurate and reproducible method for measuring liver stiffness [11]. Today, MRE has many uses, especially in detecting and staging the presence of fibrosis in the liver. It can also be used in the prediction of HCC development and decompensation in chronic liver disease, prediction of varices development in portal hypertension, and differentiation of nonalcoholic fatty liver and nonalcoholic steatohepatitis [12]. Mapping methods have been developed to determine the T1 and T2 relaxation times of tissues (T1 and T2 mapping). The main advantage of tissue mapping is the accurate determination of tissue components by revealing the relaxation time differences between tissues [13]. However, there are no studies available in the literature that evaluates changes in MRE-derived liver stiffness measure (LSM) and T1–T2 relaxation times in patients with SOS.

The aim of this study is to evaluate the role of MRE-derived liver stiffness measurement (LSM) and T1–T2 relaxation times with MRI in the diagnosis and severity assessment of SOS after oxaliplatin-based chemotherapy.

## Materials and methods

### Study population

This retrospective study was approved by the Ethics Committee of our institute. Patients who underwent liver MRI with Gd-EOB-DTPA and MRE between January 2018 and June 2020 and had received at least three cycles of oxaliplatin for colorectal, gastric, or pancreatic cancer within the six months prior to MRI were included in this study. Patients with SOS and controls without SOS findings were evaluated retrospectively. The presence of SOS was diagnosed by liver MRI (including the 20-min hepatobiliary phase) in 18 patients, while 16 patients having no SOS finding formed the control group.

Of the patients in the SOS group, 14 were treated for colon/ rectal cancer, 3 for stomach cancer, and 1 for pancreatic cancer. Of the 16 patients taking oxaliplatin who had no SOS findings, 14 were treated for colon/rectum cancer and 2 for stomach cancer. A total of thirty-four patients (M/F:22/12) were enrolled in the study.

### Laboratory findings

Alanine transaminase (ALT), aspartate aminotransferase (AST), alkaline phosphatase (ALP), gamma-glutamyltransferase (GGT), total bilirubin level, and platelet count were measured in each patient within one week of MRI. The reference ranges used by our institution for blood sample parameters were as follows: 0–33 IU/L for ALT, 0–32 IU/L for AST, 35–129 IU/L for ALP, 5–61 IU/L for GGT, 0.2–1.2 mg/dL for total bilirubin, and 150–400 × 103/μL for platelet count.

### Imaging protocol

All patients included in the study underwent liver MRI with hepatobiliary phase using Gd-EOB-DTPA (Primovist; Bayer-Schering Pharma AG, Berlin, Germany) with a 1.5-T system (Siemens AERA, Germany and GE Signa, USA) with standard body and spine matrix coils. In addition, MRE examinations were performed with 1.5 T MRI (Siemens AERA) in the same session or within two weeks after the liver MRI examination. Also, MRE, T1 and T2 mapping, T2*, and high-speed T2-corrected multi-echo (HISTO) sequences were acquired in the same session.

With the active driver generating waves at 60 Hz, the MRE was performed with a 2D-GRE sequence modified with the following parameters: repetition time (TR)/echo time (TE), 50/21 ms; flip angle: 25 degrees, bandwidth: 31.25 kHz, matrix: 256 × 128, acquisition time: 2.5 min. Depending on the liver size, 2 or 3 slices of 10 mm thickness were obtained from the largest part of the liver by holding the patient's breath.

T1 mapping was performed using the B1 inhomogeneity-corrected method with variable flip angle. Sequence parameters were as follows: repetition time (TR)/echo time (TE), 4.4/2.1 ms, rotation angle: 3 and 15 degrees, matrix: 256 × 156, FOV: 380 × 300 mm, slice thickness: 4 mm, acquisition time: 1.5 min.

T2 mapping was performed by calculating the T2 value from different TE's using the SSFP-based TrueFISP sequence and the exponential signal decay model. The sequence parameters were as follows: TR: 166 ms, TE (0 ms, 25 ms, 55 ms), flip angle: 70 degrees, FOV: 420 × 260 mm, slice thickness 10 mm, matrix: 192 × 192, NEX: 1, acquisition time: 1.2 min.

T2* mapping was performed to assess liver iron load with the following parameters: TR: 200 ms, TE: 0.93/2.14/3.35/4.56/5.77/6.98/8.19/9.4/10.61/11.82/13.03/14.24 ms, rotation angle: 20 degrees, section thickness: 10 mm, FOV: 400 × 300 mm, matrix: 160 × 85.

The HISTO sequence to assess liver fat content was performed with the following parameters: TE 12/24/36/48/72 ms, TR 3000 ms, voxel size: 30 × 30 × 30 mm, acquisition time: 15 s.

### Imaging analysis

MRI images were reviewed by two radiologists (4 and 5 years of experience) independently. First observer re-evaluated all images 6 months later for intra-reader evaluation. Discrepancies between readers were resolved by a senior radiologist with 16 years of experience. The final consensus reading was used for statistical analysis. All data were transferred to a workstation (Syngo.via, Siemens, Erlangen, Germany) for analysis. Right lobe, left lobe, and total liver stiffness scores were measured in all patients. ROIs were manually drawn during measurement, excluding lesions, large vessels, liver margins, and artifacts on the magnitude images generated with the MRE sequence. These ROIs were then copied to the stiffness maps, which provided liver stiffness values in kilopascals (kPa). The ROIs were drawn to encompass the parenchyma as much as possible.

T1 relaxation, T2 relaxation, and T2* values were measured over the corresponding sequences. Measurements were performed with a sufficiently large ROI (region of interest), excluding lesions, large vessels, liver margins, and artifacts from the right lobe of the liver (Figs. 4, 5). Corrected T1 (cT1) relaxation values were calculated using the formula “T1-420 + 20 × T2*” over T2* values [23]. Liver fat percentage was also noted.

In this study, patients who received oxaliplatin and developed SOS findings and those who received oxaliplatin but did not develop SOS findings (control group) were compared in terms of liver stiffness and T1–T2 relaxation time. Then, the patients we separated according to the severity of SOS within the SOS group were also compared with each other.

**Fig. 1:**
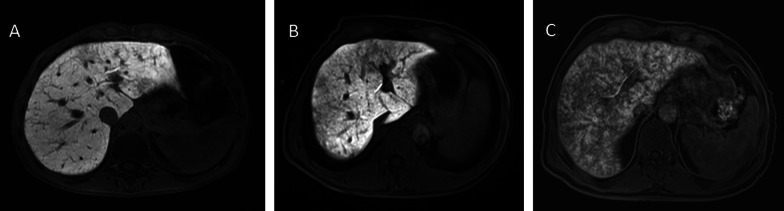
Classification of SOS severity according to the extent of reticular hypointensity on the hepatobiliary phase images; ‘mild’ (**A**) if reticular hypointensity are less than 20% of the liver parenchyma, ‘moderate’ (**B**) if 20–50%, and more than 50% ‘advance’ (**C**) grade SOS

Within the SOS group, patients were classified according to the extent of reticular hypointensity in the hepatobiliary phase. The patients with reticular hypointensity less than 20% of the liver parenchyma were classified as mild SOS, between 20 and 50% as moderate, and more than 50% as advanced SOS (Fig. 1).

### Statistical analysis

Categorical measurements were summarized as numbers and percentages and continuous measurements as mean and (minimum–maximum). When comparing continuous measurements between groups, distributions were examined, Student's *t*-test was used when variables met the parametric pretest assumption, and Mann–Whitney *U* test was used when they did not. One-way ANOVA test was used to compare continuous data by SOS severity. The accuracy of the methods was compared by calculating the areas under the curve (AUROC) from the ROC curves.

All inter- and intra-reader reliability was determined using the intra-class correlation coefficient (ICC) for continuous parameters and the kappa statistic (*κ*) and percentage agreement for categorical or binary parameters. ICC values range from 0 to 1, and values above 0.75 were considered to have excellent reliability. Kappa values range from − 1 to 1 and were categorized as poor (*κ* < 0), slight (*κ* = 0 to 0.20), fair (*κ* = 0.21 to 0.40), moderate (*κ* = 0.41 to 0.60), substantial (*κ* = 0.61 to 0.80), and almost perfect (*κ* = 0.81 to 1). The statistical significance level (*p*-value) in the tests was taken as 0.05. For statistical analysis of the data, IBM the SPSS 20.0 program was used.

## Results

### Patient demographics and laboratory findings

A total of 18 patients (M/F:11/7) were diagnosed as SOS with relevant findings on hepatobiliary phase images, and 16 patients (M/F, 11/5) were included in the control group. All observers were in agreement in terms of the presence of SOS. The mean ALT, AST, ALP, and GGT levels were higher, and platelet count was lower in SOS group in comparison with the control group with no statistically significance. The patient characteristics are summarized in Table 1.

**Table 1:** Comparison of variables in patients with SOS and control group

	Patient group (SOS)	Control group	*p* value
Age (years)	60.3 ± 10.6 60.5 (54.2–68.5)	52.9 ± 11.8 52.5 (48.2–59.7)	0.06**
Male/female	11/7	11/5	
ALT (IU/L)	33.4 ± 23.3 28 (17.7–42.5)	32.2 ± 19.5 27 (21–32.5)	1*
AST (IU/L)	41 ± 19 35.5 (28.2–54.7)	30 ± 9.8 24 (22–35.5)	0.06*
ALP (IU/L)	208 ± 158 146.5 (105–219)	135 ± 31 135 (121–159)	0.34*
GGT (IU/L)	151.4 ± 57 66.5 (33.2–128.2)	85.6 ± 24 58 (34.5–85.5)	0.57*
Total bilirubin(mg/dL)	0.96 ± 0.3 1.07 (0.68–1.26)	0.67 ± 0.2 0.64 ( 0.5–0.78)	0.052*
Thrombocyte (10^3^/μl)	174 ± 47.4 176 (139.2–213.5)	187 ± 91 200 (179.5–212.5)	0.17*
MRE-LSM (kPa)	3.14 ± 0.45 3.01 (2.85–3.35)	2.62 ± 0.5 2.55 (2.28–2.75)	0.01**
cT1 (ms)	1181 ± 151 1141 (1095–1290)	1032 ± 129 1032 (1004–1092)	0.005**
T2 (ms)	50.2 ± 3.6 49 (48–52)	44.4 ± 2.7 45 (43.5–46)	0.01**
T2 star (ms)	35 ± 4.7 33 (32–40)	33.3 ± 4.8 33 (31–38)	0.3**
Liver fat fraction (%)	4.5 ± 3 3.5 (2.7–4.7)	7.2 ± 6 6.2 (2.7–8.2)	0.26*

Statistical significant results are highlighted with bold letters

Mean ± standard deviation. Median (interquartile range)

kPa, kiloPascal

*Student’s *t* test

**Mann Whitney *U* test

### MRI findings

**Fig. 2:**
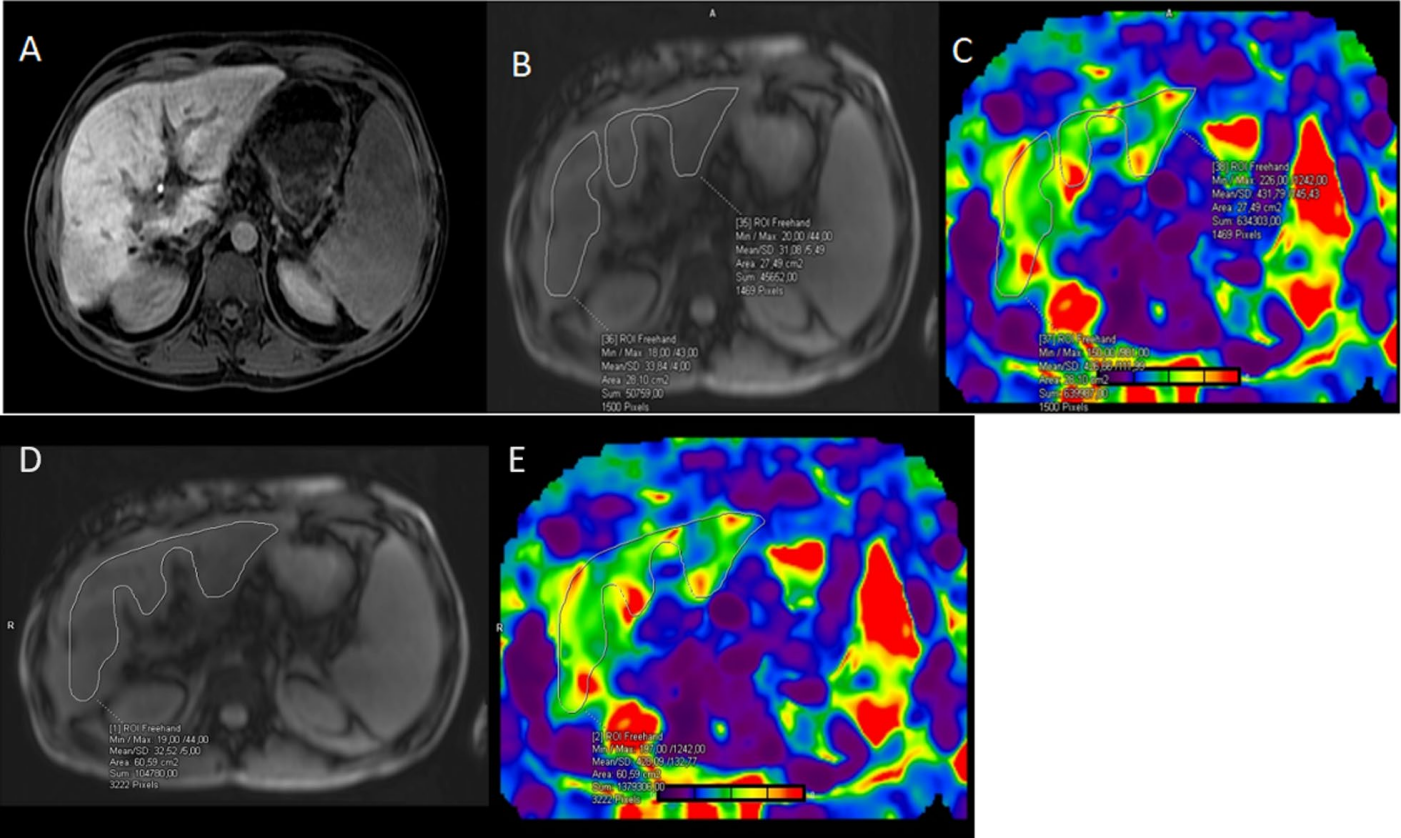
Reticular hypointensity consistent with SOS in the hepatobiliary phase image (**A**). The magnitude image of the elastogram with ROIs for the right and left lobe (**B**). The corresponding ROIs on the elastogram; right lobe: 4.26 kPa, left lobe: 4.3 kPa (**C**). The magnitude image of the elastogram with ROIs for the total liver (**D**). The corresponding ROIs on the elastogram; MRE-LSM is 4.28 kPa (**E**).

**Fig. 3:**
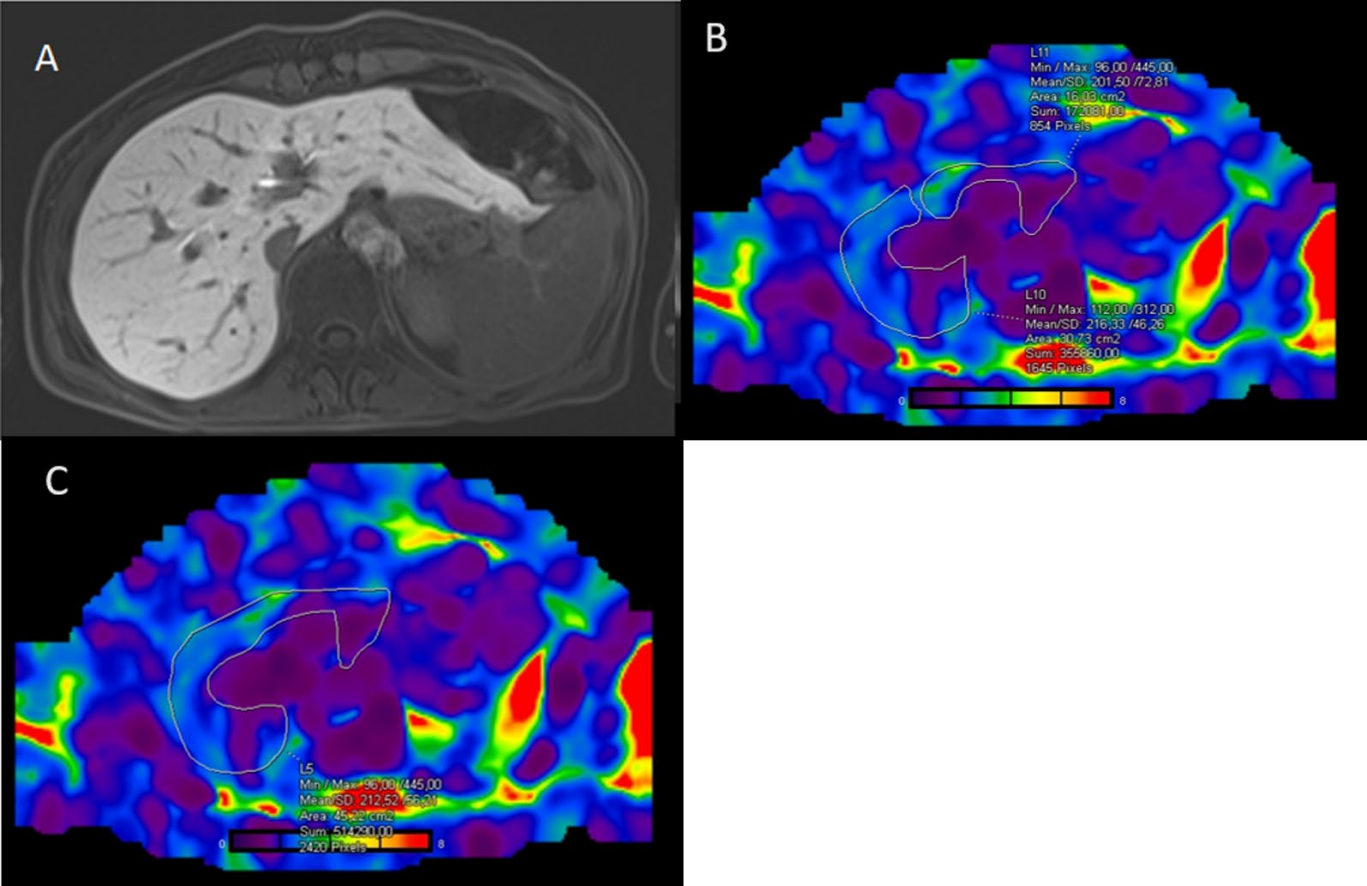
Hepatobiliary phase image in the control group case (**A**). Right lobe MRE-LSM is 2.16 kPa, left lobe MRE-LSM is 2 kPa (**B**). Total MRE-LSM is 2.12 kPa (**C**)

**Fig. 4:**
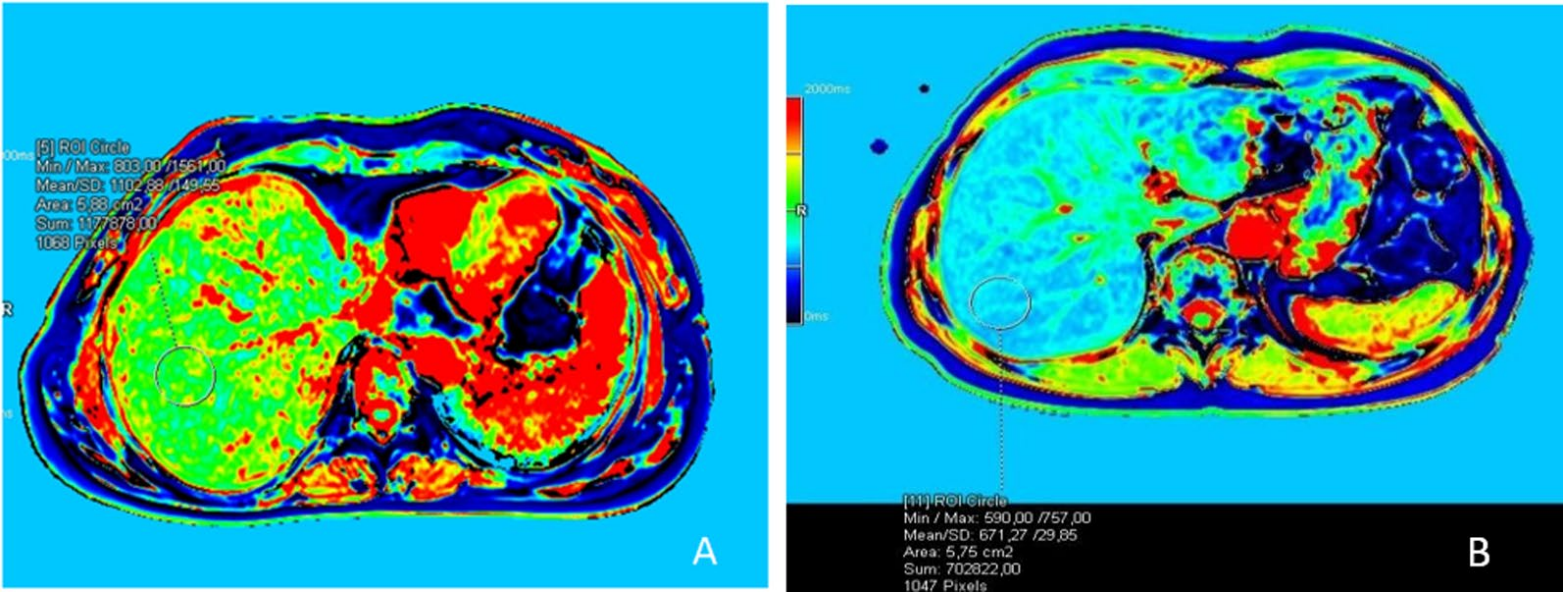
T1 mapping images demonstrates a T1 relaxation time of 1102 ms in a patient with SOS (**A**) and 671 ms in a control patient (**B**)

**Fig. 5:**
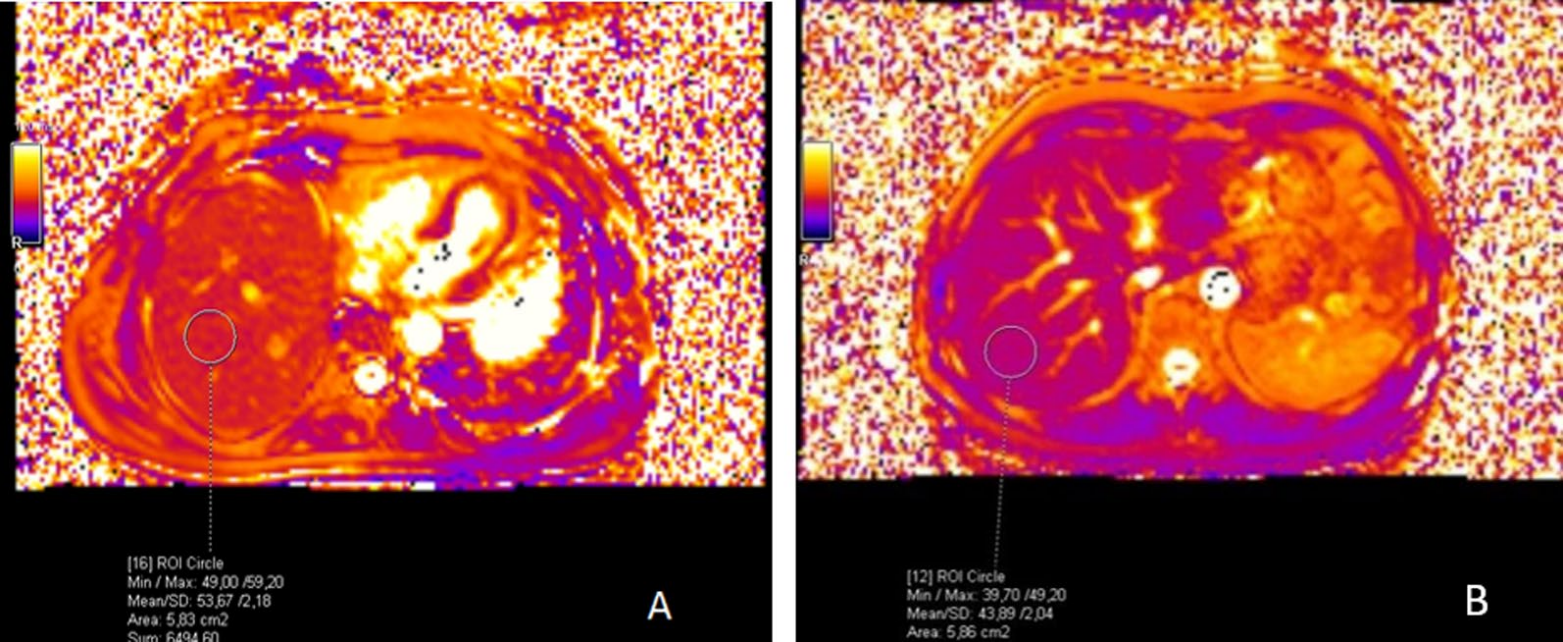
T2 mapping images of the same patients in Fig. 4 demonstrate a T2 relaxation time of 53 ms in the patient with SOS (**A**) and 44 ms in the control patient (**B**)

MRE-derived liver stiffness measurements and liver cT1, T2, T2* values in the study population are summarized in Table 1. The intra- and interobserver agreement between the two readers is summarized in Table 2. According to the results, MRE-LSM and cT1 and T2 relaxation times were significantly higher in the SOS group compared to the control group (*p* = 0.01, *p* = 0.005 ,and *p* = 0.01, respectively) (Figs. 2, 3, 4, and 5).

**Table 2:** Intra- observer and interobserver agreement of MRE-LSM, cT1, and T2 evaluation

	Intra-observer agreement		Interobserver agreement	
	ICC	95% CI	ICC	95% CI
MRE-LSM (kPa)	0.96	0.93–0.98	0.97	0.95–0.98
cT1 (ms)	0.98	0.96–0.99	0.98	0.96–0.99
T2 (ms)	0.95	0.91–0.98	0.98	0.97–0.99

ICC, intra-class correlation coefficient; CI, confidence interval

**Fig. 6:**
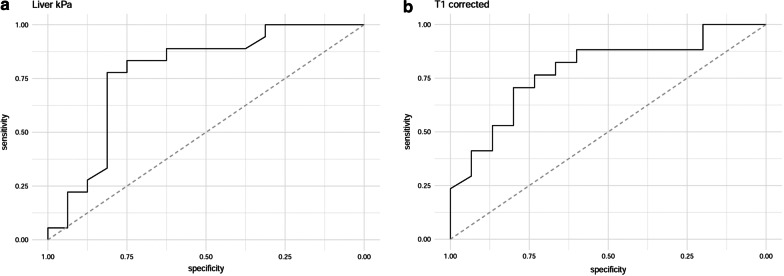
ROC curve of MRE-LSM and cT1 for differentiation of patients with SOS

The cutoff point of MRE-LSM measurement was 2.82 kPa (area under the curve, 0.88; 95% confidence interval 0.61, 0.95) to differentiate SOS from control group with a sensitivity of 78% and a specificity of 81%, and respective positive and negative predicted values of 82% and 77%. The cutoff point of cT1 was 1101 ms (area under the curve, 0.88; 95% confidence interval 0.62, 0.94) to differentiate SOS from control group with a sensitivity of 80% and a specificity of 70%, and respective positive and negative predicted values of 72% and 78% (Table 3) (Fig. 6). There was no difference between groups in T2* values reflecting iron accumulation and liver fat fraction.

**Table 3:** Diagnostic accuracy of liver stiffness and cT1 relaxation time for SOS

Patient cutoff value	AUC	Sensitivity	Specifity	PPV	NPV
MRE-LSM ≥ 2.82 kPa	0.88 (0.61–0.95)	0.78 (0.75–0.93)	0.81 (0.72–0.94)	0.82 (0.72–0.96)	0.77 (0.74–0.93)
cT1 relaxation time ≥ 1101 ms	0.88 (0.62–0.94)	0.80 (0.70–0.94)	0.70 (0.66–0.93)	0.72 (0.67–0.93)	0.78 (0.68–0.94)

Numbers in parentheses are 95% confidence interval

AUC, area under the curve; NPV, negative predictive value; PPV, positive predictive value

In SOS patients, reticular hypointensity was more dominant in the right lobe in 9 of 18 patients and in the left lobe in 2 patients on hepatobiliary phase images were evaluated. Right and left lobe dominance was equal in 7 patients. It was observed that the MRE-LSM of the right lobe was higher than the left lobe in the SOS group (*p* = 0.01). There was no difference in MRE-LSM between the right and left lobes in the control group (*p* = 0.2).

### SOS severity

**Fig. 7:**
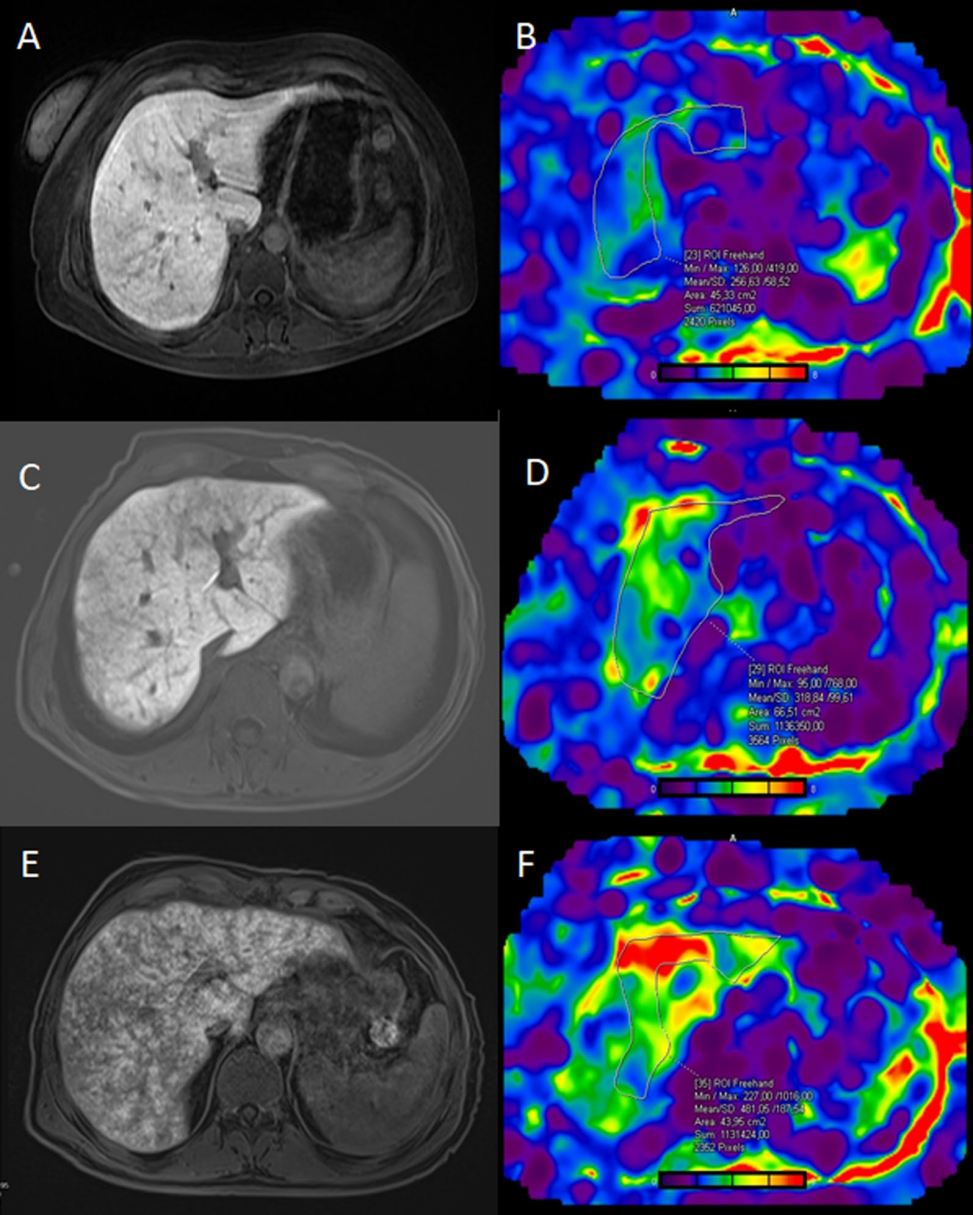
According to the amount of hypointensities on the hepatobiliary phase; Images of mild (**A**), moderate (**C**), advence (**E**) grade SOS patients. Stiffness maps of the same patients; mild (**B**), moderate (**D**), advence (**F**). As the degree of SOS increases, liver stiffness also increases. Parenchymal stiffness was measured as 2.56 kPa (**B**) in the “mild” case, 3.18 kPa (**D**) in the “moderate” case, and 4.81 kPa (**F**) in the “advance” case

Within the SOS group, patients were classified according to the extent of reticular hypointensity in the hepatobiliary phase. Three of the patients were classified as mild, ten were classified as moderate, and five were classified as advanced. The interobserver variability of the SOS severity (*κ* = 0.91; 95% CI 0.74–1) was almost perfect. The mean MRE-LSM is significantly increased with SOS severity (*p* = 0.002) (Fig. 7). cT1 and T2 relaxation times were also higher in increased SOS severity with no statistically significance (*p* = 0.325 and *p* = 0.770, respectively) (Table 4).

**Table 4:** Comparison of the relationships between SOS severity and mean liver stiffness, cT1–T2 relaxation time

	Mild SOS	Moderate SOS	Advanced SOS	*p* value
MRE-LSM (kPa)	2.8 ± 0.22 2.81 (2.48–3.07)	3 ± 0.33 2.94 (2.81–3.24)	3.65 ± 0.28 3.72 (3.39–3.89)	**0.002**
cT1 (ms)	1052 ± 82 1052 (994–1110)	1175 ± 134 1209 (1085–1290)	1245 ± 189.8 1141 (1093–1451)	0.325
T2 (ms)	48.5 ± 0.7 48.5 (48–49)	50.5 ± 4.2 49 (47.5–52.5)	50.6 ± 3.1 50 (48–53.5)	0.777

Statistical significant results are highlighted with bold letters

Mean ± standard deviation. Median (interquartile range)

After the diagnosis of SOS in our patients, oxaliplatin was removed from the chemotherapy regimen. No SOS-related liver failure or death was detected in any patient within the first 3 months after diagnosis.

## Discussion

Oxaliplatin is an important chemotherapeutic agent commonly used in neoadjuvant or adjuvant chemotherapy protocols in the treatment of GI cancer. SOS is a known complication that develops after oxaliplatin therapy. Studies have shown that the development of SOS in patients with metastatic colorectal cancer negatively affects the tumor's response to chemotherapy [4]. Although SOS is usually asymptomatic after oxaliplatin, it is associated with increased morbidity, prolonged hospital stay, increased blood transfusions, and liver failure during and after resection of liver metastases [6, 14, 15]. Therefore, identification of SOS on imaging is important to determine the timing of liver resection and to plan further chemotherapy.

Oxaliplatin-related SOS rarely causes severe impairment of liver function and is usually manifested by a mild increase in liver function tests [16]. In a study of 42 patients by Shin et al. [6], it was reported that there was no statistically significant difference between total bilirubin, ALT, AST values, and platelet counts between the SOS and control groups. In our study, no difference was found between the SOS and control group in terms of laboratory values such as total bilirubin, ALT, AST, ALP, GGT, and platelet count that enhances the role of imaging in the diagnosis of SOS in such patients.

In the present study, we observed significantly higher MRE-LSM in patients with SOS compared to control group. In the SOS group, the mean MRE-LSM of the right lobe was higher than the left lobe which is concordant with hepatobiliary phase image findings. MRE is considered the best noninvasive diagnostic tool for detecting and grading liver fibrosis. Beyond the assessment of hepatic fibrosis, MRE has potential applications in the evaluation of diffuse liver disease because hepatic parenchymal stiffness also increases in liver inflammation, congestion, mechanical cholestasis, and amyloid deposition. The reason for the increase in liver stiffness in SOS appears to be primarily due to hepatic congestion. Although fibrosis in a limited area around the central vein may develop, hepatic fibrosis is not a common feature in SOS [17]. On the other hand, previous studies using the ultrasound elastography method have shown that the increase in liver stiffness is reversible in patients with SOS [18]. Since biopsy is not performed in our study, the presence of fibrosis cannot be excluded, but it would be appropriate to evaluate whether liver stiffness is reversible by using MRE in further studies. In our study, we think that the increase in liver stiffness of SOS patients is primarily due to hepatic congestion.

In the literature, all studies that measured liver stiffness used ultrasound (US) methods such as shear wave elastography and transient elastography (TE) for the diagnosis and follow-up of SOS. Reddivalla et al. evaluated pediatric patients receiving stem cell transplantation (SCT) with US elastography and observed that follow-up US elastography at day 5 and day 14 after SCT demonstrated significantly higher liver stiffness values in patients with SOS in comparison with patients without SOS [19]. They also found that liver parenchymal stiffness began to increase nine days before the onset of clinical findings (modified Seattle criteria) in these patients. A similar study by Chollechia et al. that used TE in adult patients reported that liver parenchymal stiffness increased significantly after SCT in four patients who developed SOS compared with the group who did not develop SOS [20]. They used the European Society criteria for blood and marrow transplantation as clinical diagnostic criteria. Patients who developed SOS after bone marrow transplantation were included as a patient population in these studies. In addition, the diagnostic criteria reported to date refer to patients who develop SOS after SCT. There are no established clinical diagnostic criteria for SOS, who develop after oxaliplatin, and these patients have mild or no clinical symptoms as in the patients in our study. MRE can be used to detect clinically occult SOS in patients treated with oxaliplatin before liver surgery or to diagnose SOS in symptomatic patients at risk of developing SOS. Particularly in patients who cannot receive contrast agent, MRE can also be used for subsequent follow-up of these patients. MRE is more advantageous than the contrast-enhanced MRI and CT examinations, which are currently used to diagnose SOS because it has a relatively short imaging time and does not require the use of contrast agents. The avoidance of the effects of ionizing radiation and the possibility of quantitative measurement are further advantages.

We showed that the mean cT1 and T2 relaxation times are higher in the SOS group. According to our study, as the severity of SOS increased, MRE-LSM values also increased and MRE-LSM and SOS severity has a good correlation. However, cT1 and T2 relaxation times were found insufficient to determine the severity of SOS. T1 and T2 relaxation values obtained by tissue mapping are still investigational imaging modalities in diffuse liver disease. Although T1 and T2 relaxation values are increased in liver fibrosis, different results have been reported. For example, in a study by Hoffman et al. comparing MRE and T1–T2 relaxation values in 23 patients with chronic liver disease, MRE was found to be superior in determining fibrosis stage. They found that T1 and T2 relaxation times were moderately correlated with MRE and that combining these techniques with MRE was not superior to MRE alone [13]. In the study by Heye et al. [21], they found that T1 relaxation time was successful in distinguishing healthy patients from those with liver fibrosis, in addition to distinguishing Child–Pugh class A or B patients from class C patients. It is well known that inflammation, edema, and fibrosis in the liver prolong T1 and T2 relaxation times. To date, there is no study in the literature showing T1–T2 relaxation times of liver in SOS patients. In our study, prolonged T1 and T2 relaxation times were observed in SOS patients, similar to liver fibrosis. We think this observation could be due to hepatic congestion. In addition to the effects of T2*, iron accumulation in the liver may lead to misdiagnosis by reducing T1 relaxation time [22]. To circumvent this problem, the cT1 relaxation time is calculated by incorporating T2* value [23]. We found that the cT1 and T2 relaxation times were significantly higher in the SOS group than the control group, but didn’t reach a statistical significance.

Our study has some limitations, particularly because of its retrospective nature. The small number of patients and the differences between the groups prevent the results from being generalizable. In addition, these patients did not undergo biopsy, which is the gold standard for the diagnosis of SOS. However, dynamic liver MRI, including the hepatobiliary phase image findings, can be accepted sufficient for noninvasive SOS diagnosis [6]. In addition, this study did not consider other possible factors that may increase liver stiffness in SOS cases. Prospective studies with a larger number of patients will more clearly demonstrate the relationship between MRE mapping and SOS.

In conclusion, we observed increased MRE-LSM in patients with SOS after oxaliplatin-based chemotherapy compared to control group. There was also a good correlation with MRE-LSM and SOS severity. T1 and T2 relaxation times were also useful in diagnosing SOS, but were inadequate in determining SOS severity. We also demonstrated high intra- and interobserver agreement in evaluation of SOS presence and SOS severity with MRI as well as measurements with multiparametric MRI. MRE is particularly effective in diagnosing SOS and determining SOS severity in patients who cannot receive contrast agents, and it may be useful in the follow-up of these patients.

## Data Availability

All data were obtained from the database of our institution, based on the approval of the institutional research ethics committee.

## Abbreviations

cT1: Corrected T1
kPa: Kilopascals
LSM: Liver stiffness measurement
MRE: Magnetic resonance elastography
MRI: Magnetic resonance imaging
ROI: Region of interest
SOS: Sinusoidal obstruction syndrome
